# Introgression of the bread wheat D genome encoded *Lr34/Yr18/Sr57/Pm38/Ltn1* adult plant resistance gene into *Triticum turgidum* (durum wheat)

**DOI:** 10.1007/s00122-023-04466-z

**Published:** 2023-10-17

**Authors:** Hongyu Li, Peng Zhang, Ming Luo, Mohammad Hoque, Soma Chakraborty, Brenton Brooks, Jianbo Li, Smriti Singh, Kerrie Forest, Allan Binney, Lianquan Zhang, Diane Mather, Michael Ayliffe

**Affiliations:** 1 CSIRO Agriculture and Food, Clunies Ross Street, GPO Box 1700, Canberra, ACT 2601 Australia; 2 Plant Breeding Institute, School of Life and Environmental Sciences, University of Sydney, Cobbitty, NSW 2570 Australia; 3 Agriculture Victoria, Department of Energy, Environment and Climate Action, AgriBio Centre for AgriBioscience, 5 Ring Rd, Bundoora, VIC 3083 Australia; 4 Triticeae Research Institute, Sichuan Agricultural University, 211 Huimin Road, Wenjiang, Chengdu, 611130 Sichuan China; 5 School of Agriculture, Food & Wine, The University of Adelaide, PMB 1, Glen Osmond, SA 5064 Australia

## Abstract

**Key Message:**

Lack of function of a D-genome adult plant resistance gene upon introgression into durum wheat.

**Abstract:**

The wheat *Lr34/Yr18/Sr57/Pm38/Ltn1* adult plant resistance gene (*Lr34*), located on chromosome arm 7DS, provides broad spectrum, partial, adult plant resistance to leaf rust, stripe rust, stem rust and powdery mildew. It has been used extensively in hexaploid bread wheat (AABBDD) and conferred durable resistance for many decades. These same diseases also occur on cultivated tetraploid durum wheat and emmer wheat but transfer of D genome sequences to those subspecies is restricted due to very limited intergenomic recombination. Herein we have introgressed the *Lr34* gene into chromosome 7A of durum wheat. Durum chromosome substitution line Langdon 7D(7A) was crossed to Cappelli *ph1c*, a mutant derivative of durum cultivar Cappelli homozygous for a deletion of the chromosome pairing locus *Ph1*. Screening of BC_1_F_2_ plants and their progeny by KASP and PCR markers, 90 K SNP genotyping and cytology identified 7A chromosomes containing small chromosome 7D fragments encoding *Lr34*. However, in contrast to previous transgenesis experiments in durum wheat, resistance to wheat stripe rust was not observed in either Cappelli/Langdon 7D(7A) or Bansi durum plants carrying this *Lr34* encoding segment due to low levels of *Lr34* gene expression. Key message

**Supplementary Information:**

The online version contains supplementary material available at 10.1007/s00122-023-04466-z.

## Introduction

Diseases known as rusts remain a constant threat to global production of both hexaploid bread wheat (*Triticum aestivum* L., 2n = 6x = 42) and tetraploid durum and cultivated emmer wheat (*Triticum turgidum* subsp. *durum* (Desf) Husn. and *T. turgidum* subsp. *dicoccum* (Schrank ex Schübl.) Thell., 2n = 4x = 28) (Kolmer 2005, 2015; Singh et al. 2011; Huerta-Espino et al. 2011; Bhavani et al. 2019). Two forms of resistance are commonly employed to help protect these crops from rusts, all stage resistance (ASR) and adult plant resistance (APR) (Singh et al. 2015; Ayliffe et al. 2022). ASR, also known as seedling resistance, can provide high levels of resistance but is generally only effective against a single pathogen species and usually race specific. While individual ASR genes can be very effective, they generally suffer from a lack of durability and are often rapidly overcome by pathogen mutation (Ayliffe et al. 2008).

In contrast, some (but not all) APR genes provide broad spectrum resistance that is generally partial and, in some instances, effective against multiple pathogen species. One such examples is the *Lr34/Yr18/Sr57/Pm38/Ltn1* APR gene (hereafter referred to as *Lr34*) which has provided broad spectrum, partial resistance to leaf rust, stripe rust, stem rust and powdery mildew (Dyck et al. 1966; Kolmer et al. 2008; Herrera-Foessel et al. 2014). *Lr34* encodes an ABC transporter (Krattinger et al. 2009) that is suggested to transport abscisic acid (Krattinger et al. 2019), although the exact mechanistic basis of resistance remains unclear. A remarkable feature of *Lr34* is its durability (Dyck et al. 1966; Kolmer et al. 2008). It has been used for many decades in many wheat cultivars and remains durable, despite large scale deployment and constant rust pathogen evolution that continues to overcome many ASR genes (Singh et al. 2011; Olivera et al. 2015; Patpour et al. 2017).

*Lr34* is encoded on chromosome arm 7DS of bread wheat thereby making this valuable gene unavailable for deployment in tetraploid wheat by standard conventional breeding. Previously *Lr34* was introduced into durum wheat cultivar Stewart by *Agrobacterium*-mediated transformation (Rinaldo et al. 2017). Interestingly, these transgenic plants showed seedling resistance to rust diseases at ambient temperature which is atypical for this APR gene. Those transgenic durum plants with the highest levels of *Lr34* expression showed the greatest resistance (Rinaldo et al. 2017). RNA analysis also showed that these resistant seedlings had elevated *Lr34* expression compared with wild type bread wheat seedlings carrying *Lr34*. This increased expression was ascribed to the different genomic locations in which the transgene was integrated. These data confirmed that, with appropriate expression levels, *Lr34* is functional in durum cultivar Stewart and no additional genes encoded on the wheat D genome are required for this seedling resistance. It is noteworthy that *Lr34* is also functional in heterologous hosts such as maize, rice, barley and sorghum, albeit with pleiotropic effects associated with higher expression levels (Risk et al. 2013; Krattinger et al. 2016; Schnippenkoetter et al. 2017; Sucher et al. 2017).

In this study, we attempted to make the *Lr34* gene available for durum breeding using a nonGM deployment strategy. A mutation in the *Ph1* locus (*ph1c*) was used to promote homoeologous recombination (Sears 1977; Giorgi and Barbera 1981) in durum wheat between chromosome 7A and a 7D chromosome encoding *Lr34*. While this was successfully achieved, in contrast to previous transgenic experiments (Rinaldo et al. 2017), *Lr34* resistance was not observed in plants carrying *Lr34* on 7A/7D chromosomes. Expression analysis of field grown materials showed that *Lr34* expression in these durum plants was significantly reduced when compared with bread wheat controls. Given the clear correlation between *Lr34* resistance and gene expression levels observed in transgenic durum wheat (Rinaldo et al. 2017), this reduced expression likely explains the inability of this gene to provide resistance in this heterologous chromosome context.

## Materials and methods

### Germplasm

A durum wheat disomic substitution line containing a chromosome 7D substitution for chromosome 7A in cultivar Langdon (LDN), LDN 7D(7A) (Joppa and Williams 1988) was used as a parent. Chromosome 7D in this line was derived from bread wheat cultivar Chinese Spring (CS) which carries *Lr34*. The second parent was Cappelli *ph1c*, a durum wheat line that is homozygous for a deletion of the *Ph1* locus (Giorgi and Barberra 1981). Cappelli/LDN 7D(7A) derived plants containing small 7D introgressions encoding *Lr34* were tested for stripe rust resistance in field trials. These Cappelli/LDN 7D(7A) plants were also crossed for three generations using Bansi, an Indian landrace that is highly susceptible to Australian *Puccinia striiformis* f. sp. *tritici* Eriks. and Henn. (*Pst*) isolates, as a recurrent parent with homozygous derivatives of this material named Bansi *Lr34*. They were also backcrossed for six generations to Italian cultivar Svevo and homozygous progeny lines selected from selfed BC_6_ individuals to create Svevo *Lr34*. Both Bansi *Lr34* and Svevo *Lr34* was field trialled for resistance to stripe rust.

### Plant growth conditions

Glasshouse plants were grown with a 16 h, 22 °C/8 h, 18 °C growth temperature regime in 25 cm pots containing a compost/soil mixture and fertilised by periodic application of osmocote slow-release fertiliser (Scotts, Australia). Plants grown in growth cabinets had a 16-h light/8-h dark photoperiod and 22 °C/16 °C temperature cycle. For speed breeding applications plants were grown in 15 cm pots in growth cabinets under a 22-h light/2-h dark photoperiod at a constant 22 °C. Speed breeding was used to backcross *Lr34* introgressions from LDN 7D(7A)/Cappelli progeny to both Bansi and Svevo.

### Field trials

Field trials were undertaken at the experimental sites, Horse Research Unit and Lansdowne, of the Plant Breeding Institute, University of Sydney, Cobbitty, NSW in 2021 and 2022, respectively. Plants were grown in replicated 1-m rows with seeds sowed 10 cm part. A mixture of susceptible cultivars (spreader) was planted every six rows to facilitate disease development and spread. Susceptible pots from the glasshouse were put in the field to initiate the rust development. Plants were infected at the 5-leaf stage with a mixed inoculum, consisting of predominant local *Pst* pathotypes. Experimental material was infected naturally throughout the growing season from uredinospores on adjacent spreader rows. Flag leaves were scored for adult plant resistance when the susceptible cultivar Bansi showed an 8–9 response on a 1–9 (very resistant to very susceptible) scale (Bariana et al. 2007).

### Quantification of fungal biomass

Flag leaves (> 5) from plants grown in field disease nurseries were harvested just prior to senescence, weighed and processed as described by Ayliffe et al. (2013). Briefly, samples were autoclaved in 1 M KOH and then neutralised in 50 mM Tris pH 7.5. Samples were then homogenised with a sonicator and resuspended at 200 mg/ml in Tris buffer. Aliquots (200 μl) were stained with 10 μg of wheat germ agglutinin FITC for 30 min, washed 3 times and finally resuspended in 50 mM Tris buffer. Fluorometric measurements were made with a Wallac Victor 1420 multilabel counter (Perkin-Elmer Life Science, Waltham, MA, USA) fluorometer with 485-nm adsorption and 535-nm emission wavelengths and a 1.0-s measurement time. Triplicate reactions were undertaken for each sample.

### DNA extraction for PCR analyses

Plant DNA was extracted from freeze dried tissue using a Nimbus liquid handling robot (Hamilton, Province, USA). Freeze dried leaf tissue was macerated with a ball bearing and heat treated for 1 h in extraction buffer (0.1 M Tris pH 8.0, 0.05 M EDTA, 1.25% SDS). After the addition of 6 M NH_3_Ac and centrifugation the supernatant was precipitated with isopropanol and DNA washed in 70% ethanol and finally resuspended in TE buffer (10 mM Tris pH 8.0, 1 mM EDTA).

### DNA quantitative PCR analysis

DNA samples for quantitative DNA analysis of the wheat D genome specific repeat sequence, *Dgas44* (Bryan et al. 1998; McNeil et al. 1994; Han et al. 2014), were amplified with GoTaq DNA polymerase (Promega) using a CFX96 Real Time System and C100 Touch Thermo Cycler (Bio‐Rad). *Dgas44* amplification products were normalized relative to wheat 28S rRNA genes (Han et al. 2016; GenBank AY049041.1). The comparative C_T_ method was used for quantification (Schmittgen and Livak 2008) with three replicate reactions per sample. *Dgas44* and 28S rRNA specific primers are listed in Table S1.

### KASP analysis of single-nucleotide and presence–absence polymorphisms

Six single-nucleotide polymorphisms (SNPs) between sequences on chromosome 7D of bread wheat and the homeologous sequences on chromosome 7A of durum wheat were assayed on 71 progeny plants using Kompetitive Allele-specific PCR (KASP; LGC/Biosearch, UK) primer sets (Table S1). Genotyping was performed on a SNPline platform (LGC/Biosearch, UK), according to the manufacturer’s instructions. Similarly, KASP primer sets (Table S1) were used to detect the presence of the chromosome-7D bread wheat genes *Lr34* (primer set Lr34_TCCIND, Rasheed et al. 2016) and *Xat* (GenBank MN233788; Watkins et al. 2019), neither of which is present in durum wheat.

### Gel-based analysis of presence–absence and length polymorphisms

Chromosome 7D markers were generated by comparing random regions of CS chromosome 7D sequence to chromosome 7A to identify potential deletion polymorphisms. Primers were then designed to either amplify products only from chromosome 7D or amplify products of different lengths from chromosomes 7D and 7A. PCR products were amplified from CS using a Phire Plant Direct PCR Kit (Thermo Scientific, USA), resolved by 1% agarose gel electrophoresis and sequenced by Sanger sequencing to confirm their identities. Primer pairs were then screened against LDN and LDN 7D(7A) to identify 7D-specific markers. Four markers (Cs7D1, Cs7D2, Cs7D3 and Cs7D4) identified by this approach (Table S1) were selected for genotyping of progeny plants. The presence of *Lr34* in germplasm was also determined using dominant gene specific PCR amplification primers, ABCTF4N (Krattinger et al. 2009) and Lr34 PLUSR (Lagudah et al. 2009), derived from the cloned *Lr34* gene sequence.

### Array-based analysis of presence–absence polymorphisms

An iSelect 90 K wheat SNP genotyping array developed by Wang et al. (2014) was used to assess the presence or absence of fluorescent signals from probes known to be specific to chromosomes 7A, 7B or 7D of bread wheat.

### RNA qPCR analysis

RNA for quantitative PCR analysis was extracted from wheat flag leaves of plants grown in the field. A Spectrum Plant Total RNA Kit (Sigma-Aldrich) was used for RNA extraction and On-column DNase I Digestion Kit used to remove genomic DNA (Sigma-Aldrich). cDNA was produced by reverse transcription using a Phusion RT-PCR kit (Finnzymes) and qPCR undertaken using a CFX96 Real Time System and C1000 Touch Thermo Cycler (Bio‐Rad). *Lr34* transcripts were normalized relative to the wheat *glyceraldehyde 3-phosphate dehydrogenase* gene (*TaGAPDH;* GenBank AF251217) using the comparative C_T_ method (Schmittgen and Livak 2008). Primer sequences are shown in Table S1.

### DNA blot analysis

DNA from durum seedlings was isolated using the CTAB method (Saghi-Maroof et al. 1984). DNAs were digested with *EcoRI*, prior to 0.8% agarose gel electrophoresis and then transferred to nylon membranes and hybridized as described by Collins et al. (1998). The *Lr34* DNA probe was PCR amplified using primers listed in Table S1 to produce a 372 bp product that encompassed nucleotides 64,619–64,240 of the *Lr34* genomic sequence (GenBank FJ436983.1). The probe was labeled with [P^32^]-dCTP using a Megaprime DNA labelling system (Amersham).

### Cytogenetic analysis

Fluorescence in situ hybridisation (FISH) and genomic in situ hybridisation (GISH) analyses were undertaken on plant root tip cells as previously described (Zhang et al. 2001, 2004; Lang et al. 2018). A, B and D genome chromosomes were distinguished in GISH analysis using fluorescein-12dUTP (fluorescent green colour) (Roche Diagnostics Gmbh, Mannheim, Germany) and tetramethyl-rhodamine-5-dUTP (red colour) labelled probes derived from total genomic DNA of *T. urartu* and *Aegilops tauschii*, respectively. Individual chromosomes were identified by FISH analysis with oligonucleotide probes, oligo-pSc119.2-1 (green) and oligo-pTa535-1 (red) (Tang et al. 2014).

## Results

**Fig. 1:**
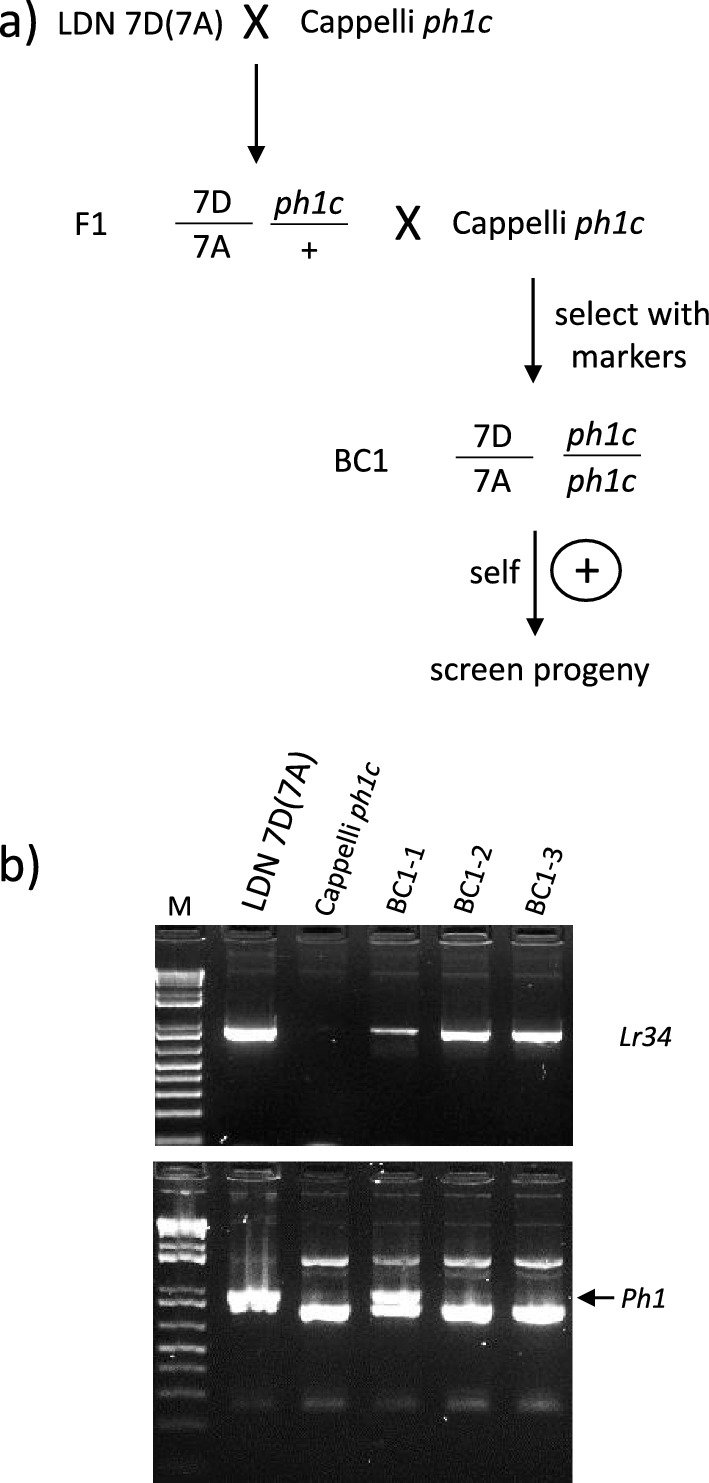
**a** Crossing strategy used for introgression of the bread wheat chromosome 7D region that encodes *Lr34* gene into chromosome 7A of durum wheat. **b** Molecular marker analysis of BC_1_ plants produced by crossing LDN 7D(7A) plants with Cappelli *ph1c* and using Cappelli *ph1c* as a recurrent parent. The upper panel depicts PCR products obtained with a dominant *Lr34* PCR marker derived from the cloned *Lr34* gene sequence and amplified using primers ABCTF4N (Krattinger et al. 2009) and Lr34 PLUSR (Lagudah et al. 2009), while the lower panel shows gel electrophoresis of products obtained for a dominant PCR marker specific for the wild type *Ph1* gene. This latter marker also amplified nonspecific products in the Cappelli *ph1c* deletion mutant. Each panel shows amplification products from LDN 7D(7A) (lane 2), Cappelli *ph1c* (lane 3) and BC_1_ plants (lanes 4–6). A 1 kb Plus molecular weight DNA marker (M) (Invitrogen) is shown in lane 1

Tetraploid wheat chromosome substitution line LDN 7D(7A) was crossed to Cappelli *ph1c* (Riley and Chapman 1958; Giorgi and Barberra 1981), and a crossing and screening strategy similar to that reported by Han et al. (2016) was undertaken (Fig. 1a). Chromosome 7D present in LDN 7D(7A) was derived from the hexaploid wheat cultivar Chinese Spring which encodes the *Lr34* gene (Joppa and Williams 1988). F_1_ plants were backcrossed to the Cappelli *ph1c* mutant and BC_1_ plants identified that were, firstly homozygous for *ph1c* as evidenced by the absence of a wild type *Ph1* PCR product (Wang et al. 2002) and secondly, contained chromosome 7D as indicated by an *Lr34* marker derived from the cloned *Lr34* gene sequence (Table S1) (Fig. 1b) (Lagudah et al. 2009). Progenies (BC_1_F_2_) were generated from two BC_1_ plants by self-fertilisation with 101 progeny from the first BC_1_ plant and 81 progeny from the second BC_1_ plant subsequently analysed.

**Fig. 2:**
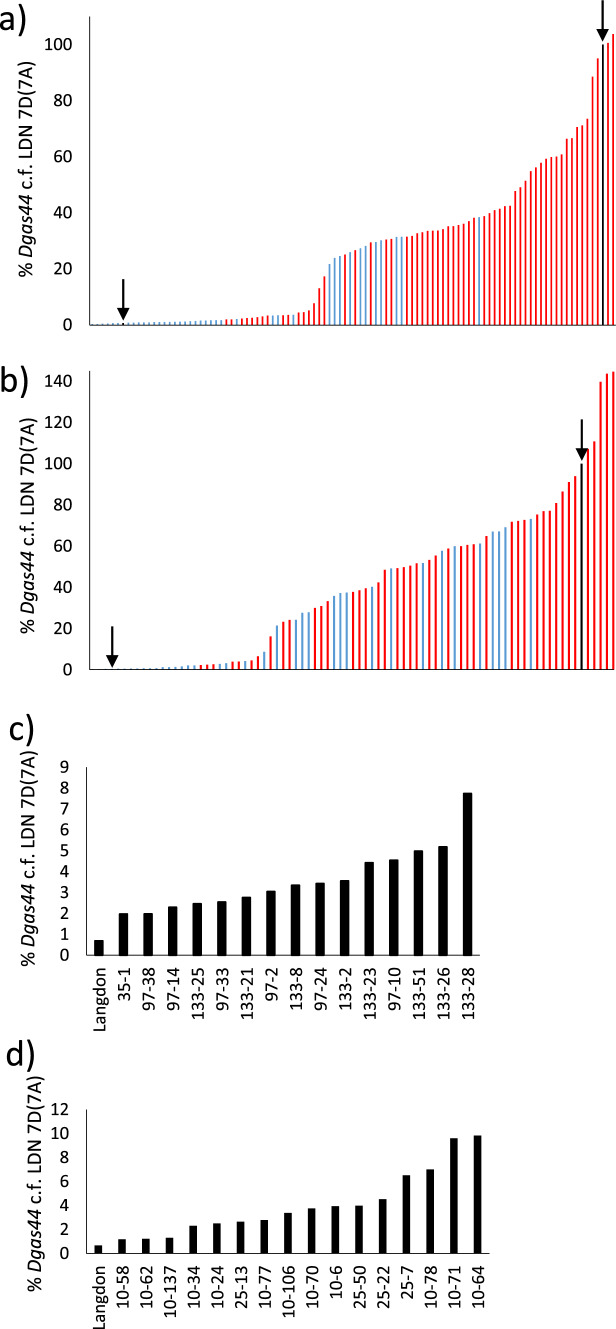
**a** Quantification of a D genome-specific repeat sequence, *Dgas44*, in LDN 7D(7A) x Cappelli *ph1c* BC_1_F_2_ progeny from BC1 plant 1 using qPCR. Each column depicts the relative amount of *Dgas44* amplification product obtained from a single progeny plant. *Dgas44* amplification products were normalised relative to a control wheat gene (*GAPDH*) and this ratio was then normalised relative to the *Dgas44*/*GAPDH* value obtained for LDN 7D(7A). The LDN 7D(7A) value (100%) is shown as a vertical black column that is arrowed on the right whereas the value obtained for a LDN control line is highlighted by an arrow on the left. Red lines indicate plants containing *Lr34* and blue lines show plants without *Lr34*. **b** Graph depicting the same *Dgas44* assay shown in (A) undertaken on progeny from BC1 plant 2. **c** Graph of the same plants shown in (A) that contain *Lr34* and low amounts of *Dgas* sequence. **d** Graph of the same plants shown in (B) containing *Lr34* and low amounts of *Dgas44* sequence (colour figure online)

As a preliminary screen DNAs from BC_1_F_2_ plants were subject to real time qPCR whereby a wheat D genome-specific repeat sequence, *Dgas44* (McNeil et al. 1994) was quantified relative to the wheat 28S *rRNA* genes (Han et al. 2016) (Fig. 2a, b). These plant DNAs were also screened for the presence of *Lr34* using the marker described above (Fig. 1b; Table S1). From this analysis, 15 and 16 *Lr34* positive plants were identified from each cross (14.8% and 19.7% of plants screened, respectively) that contained 2–10% of *Dgas44* sequences compared with the homozygous LDN 7D(7A) parent substitution line (Fig. 2c, d). Potentially these plants contained small 7D introgressions in chromosome 7A that encode *Lr34*. Given the relatively large number of potential recombinants identified in these two families only progeny from the first family were analysed further as these plants were planted earlier and hence more mature.

To confirm the results obtained from *Dgas44* qPCR a series of dominant and co-dominant KASP markers were used to screen 71 BC_1_F_2_ plants from this family. These markers confirmed that frequent homoeologous recombination events occurred between chromosomes 7A and 7D in the *ph1c* mutant background (Fig. S1). From the analysis of these 71 plants, 46 (65%) contained at least one recombinant 7D/7A chromosome (Fig. S1). Of the 15 plants originally identified as *Lr34* positive and containing less than 10% *Dgas44* (Fig. 1c), 11 underwent this KASP analysis and all were confirmed to have small 7D introgressions (Fig. S1, marked with a black asterisk).

A set of 16 *Lr34* positive plants were selected for further analysis. These included 11 of the 15 plants that had *Dgas44* levels below 10% (Fig. 2c) and for which supporting KASP marker data was successfully obtained (Fig. S1, black asterisk). Plant 43-2 was included, which contained 13% *Dgas44* and only three of 16 7D KASP alleles (Fig. S1, marked with X). Finally, four plants (97-4, 97-5, 97-17, 133-27) were also further analysed as they potentially contained a chromosome with a small *Lr34* introgression, in addition to a complete 7D chromosome (Fig. S1, red asterisks).

**Fig. 3:**
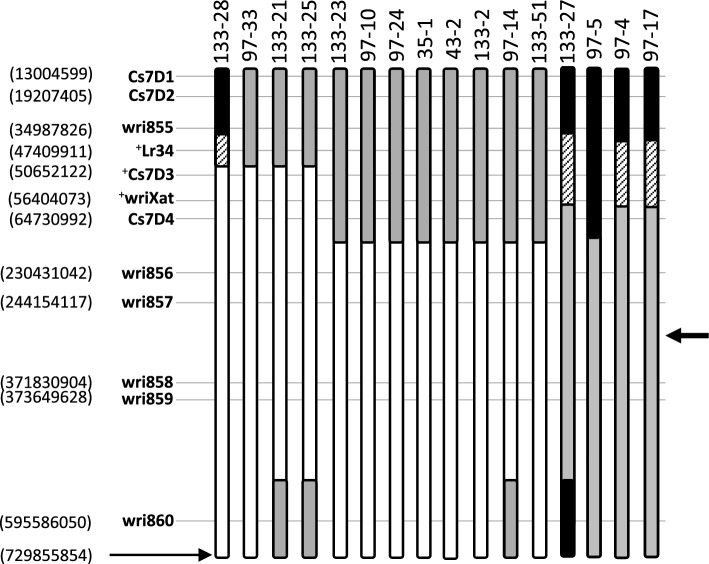
Mapping of chromosome 7D introgressions on chromosome 7A. KASP and PCR markers used to define 7D introgressions in BC_1_F_2_ plants are indicated on the left-hand side with nucleotide co-ordinates of each sequence in the CS genome v1.0 shown in parenthesis. Chromosome 7A from plants (labelled at top) are shown diagrammatically (not to scale) with homozygous 7A sequences white, heterozygous 7D/7A sequences grey and homozygous 7D sequences black. The four plants on the right (137-27, 97-5, 97-4 and 97-17) contained a full length 7D chromosome in addition to a recombinant 7D/7A introgression chromosome. The hatched region shown for plants 133-28, 137-27, 97-4 and 97-17 indicate that recombination break points that occurred in this region could not be more accurately determined due the absence of co-dominant 7D/7A markers in the region. Zygosity of dominant markers for the remaining chromosomes was assumed based on the genotypes of surrounding co-dominant markers, a strategy consistent with the least number of independent recombination events having occurred. Dominant markers are prefaced with (+). The arrow at right indicates approximate centromere position

Four 7DS PCR markers that resolved LDN 7D(7A) from both LDN and Cappelli *ph1c* were identified as described in the materials and methods. Three of these markers (Cs7D1, Cs7D2, Cs7D4) were codominant and one dominant (Cs7D3) (Fig. 3a). These markers were screened on the 16 plants described above and the PCR and KASP marker data combined for genotyping. Four plants (97-33, 133-21, 133-25 and 133-28) had similar sized introgressions carrying *Lr34* and contained less than 8% of chromosome 7D based upon physical marker positions and the CS reference genome sequence v1.0. Of these plants, one (133-28) was homozygous for three co-dominant 7D markers (wri855, Cs7D1 and Cs7D2) indicating it had inherited two recombinant chromosomes, although the zygosity for *Lr34* could not be established given the dominant nature of this marker. Two of these five plants (133-21 and 133-25) also contained a 7D marker at the terminus of the long arm of 7A indicating they contained either two recombinant chromosomes or one with two introgressions.

A further eight plants (35-1, 43-2, 97-10, 97-14, 97-24, 133-2, 133-23, 133-51) were heterozygous for similar sized 7D introgressions that encoded less than 31% of chromosome 7D with one plant also containing the distal end of 7DL (Fig. 3, 97-14). Of the four plants (97-4, 97-5, 97-17 and 137-27) that contained a complete chromosome 7D in addition to a 7D/7A recombinant one (Fig. 3) plant 97-5 was likely homozygous for *Lr34*, whereas the recombination break point and *Lr34* zygosity could not be determined for the other three plants due to a lack of co-dominant markers in the region (Fig. 3).

**Fig. 4:**
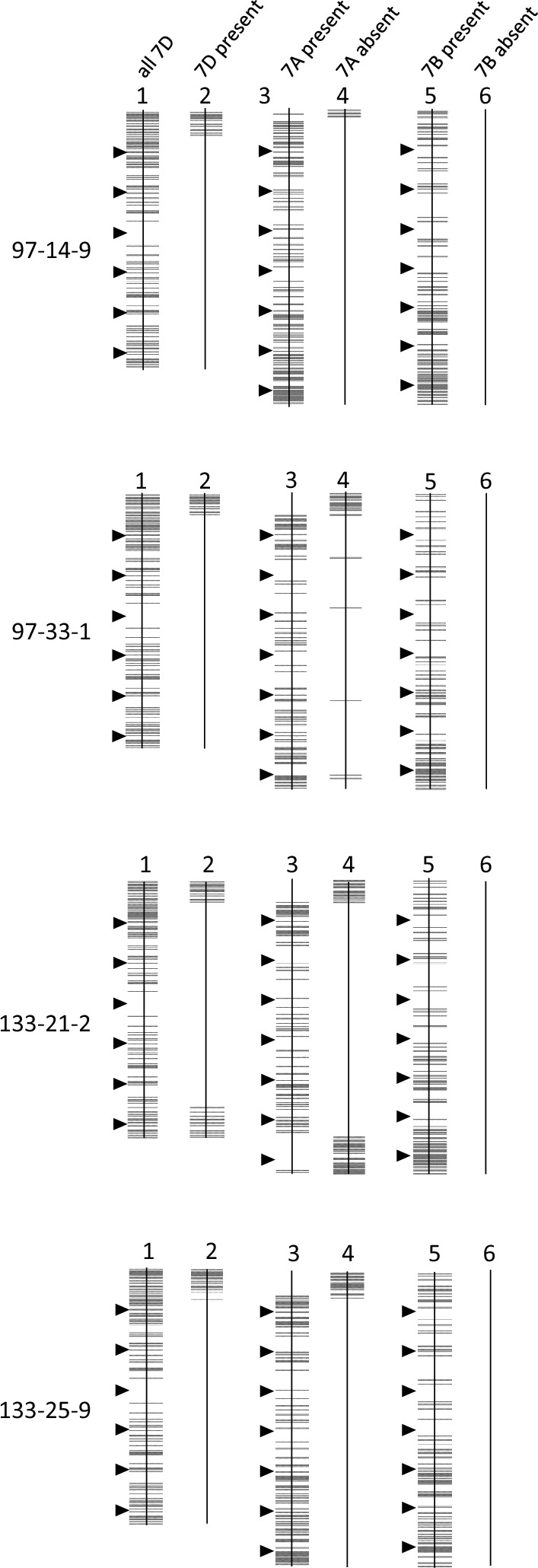
90 K SNP marker analysis of 7D and 7A chromosomes in selected homozygous BC_1_F_3_ plants (97-14-9, 97-33-1, 133-21-2, 133-25-9). Markers are shown as grey lines, or boxes when groups of markers are present, on each chromosome with CS v1.0 genome nucleotide co-ordinates shown as arrowheads on the left of each chromosome pair with each arrowhead depicting 100 Mb intervals. Karyotypes 1–6 are as follows; (1) 7D specific markers present on chromosome 7D of the parental LDN 7D(7A) substitution line (all 7D), (2) 7D markers present in the BC1F3 plants indicated (7D present), (3) 7A markers present in the BC1F3 plants (7A present), (4) 7A markers absent in BC1F3 plants (7A absent), (5) 7B markers present in BC1F3 plants (7B present), (6) 7B markers absent in the BC1F3 plants (7B absent)

Four heterozygous BC_1_F_2_ plants that carried *Lr34* (97-14, 97-33, 133-21, 133-25) were self-fertilised and homozygous BC1F3 seedlings selected using codominant markers Cs7D1, Cs7D2 and Cs7D4 (Fig. 3; Table S1). DNAs from these four homozygous progenies (97-14-9, 97-33-1, 133-21-2, 133-25-9) were then assayed using wheat Infinium iSelect 90 K SNP arrays to further resolve both the size of introgressed 7D segments and the number of introgressions within the chromosome. SNP markers were identified that were polymorphic between the LDN and Cappelli *ph1c* genomes and also a set that was specific to the LDN 7D(7A) chromosome substitution line (i.e., 7D specific) (Fig. 4).

Chromosome 7D specific markers were identified in these plant DNAs that further confirmed the presence of 7D introgressions (Fig. 4) and were generally consistent with the initial marker mapping results (compare Figs. 3 and 4). Evidence for homozygosity and the presence of 7DS introgessions in plants 97-33-1, 133-21-2 and 133-25-9 was supported by the absence of 7A markers located in introgressed 7DS regions and presence of 7A markers on the remainder of the chromosome (Fig. 4). This SNP marker pattern showed slightly less correlation for plant 97-14-9 for unknown, presumably technical reasons. No evidence of introgressions into chromosome 7B for any of the plants analysed was observed (Fig. 4). Plant 133-21-2 was homozygous for a double recombinant chromosome with 7D introgressions present at both ends of 7A, given the absence of 7A markers in the corresponding regions (Fig. 4). Finally, 90 K SNP analysis (Fig. 4) showed that plants 97-14-9 and 133-25-9 were both homozygous for a recombinant chromosome containing only a single 7DS integration suggesting their parents were likely to contain two separate recombinant chromosomes rather than a single recombinant chromosome with two 7D introgressions (Fig. 4).

**Fig. 5:**
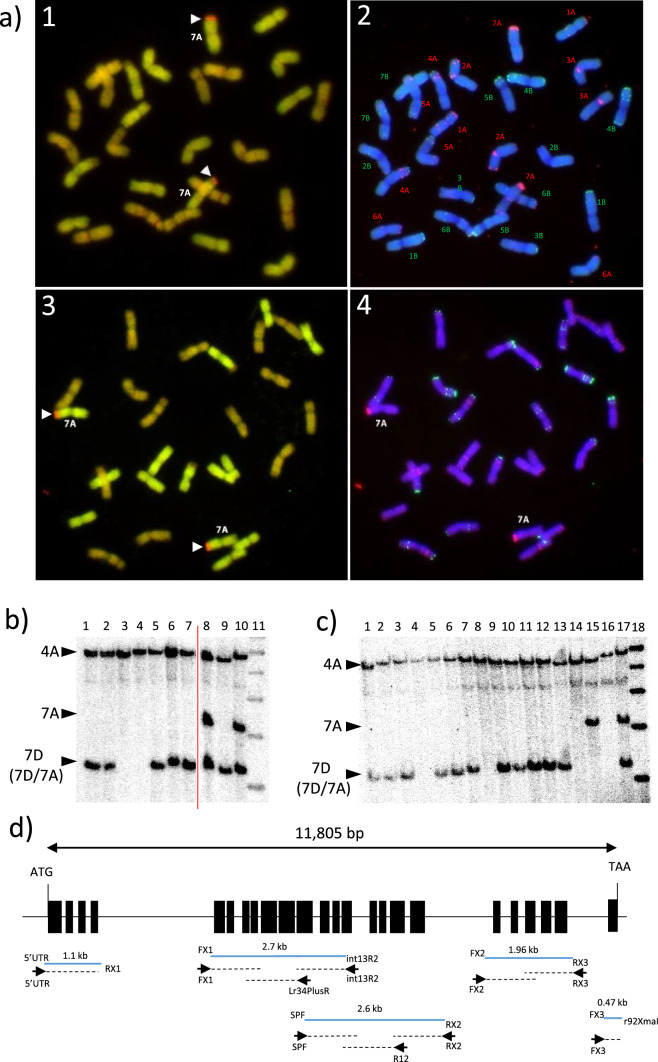
Cytogenetic and DNA blot analyses of *Lr34* durum wheat plants. **a** Cytological analysis of root tip chromosome spreads from two BC_1_F_3_ plants, 97-14-3 (upper panels) and 97-33-1 (lower panels). GISH chromosomes on the left were hybridised with A and D genome specific probes generating green and red signals, respectively, whereas FISH images on the right were from hybridisation with oligo-pTA535-1 (red label) and pSc119.2-1 (green label) to identify individual chromosomes. Arrow heads indicate introgressed chromosome 7D segments that show red fluorescence in GISH images (1 and 3). **b**, **c** Segregation of the *Lr34* gene in progeny of Bansi *Lr34*-13 and Bansi *Lr34*-15 plants. DNAs were digested with *Eco*RI and hybridised with a *Lr34* probe. Arrow heads at the left of each figure indicate the chromosome origin of each restriction fragment based upon the CS genome sequence v1.0. Note that the 7B homoeologue of *Lr34* is thought to have translocated to chromosome 4A (Krattinger et al. 2011). In panel (B), lanes 1–7 contain DNAs from progeny of Bansi plant *Lr34*-13 while lanes 8–10 contain genomic DNAs of bread wheat cv. Fielder, durum chromosome substitution line LDN 7D(7A) and bread wheat cv. Robin, respectively. In panel (C) lanes 1–12 contain DNAs from progeny of line Bansi Lr34-15, whereas lanes 13–17 contain genomic DNAs from chromosome substitution line LDN 7D(7A), wild type LDN, Cappelli *ph1c* durum, durum cv. Bansi and bread wheat cv. Robin, respectively. Note a presence/absence polymorphism for the 7A *Lr34* RFLP fragment exists between durum lines Cappelli *ph1c*, LDN and Bansi which is described further in Fig. S3. In panels (B) and (C) lanes 11 and 18, respectively, show molecular weights of 6, 5, 4, 3 and 2 kb. The red line in panel (B) indicates the point at which additional lanes on the same autoradiograph were removed as they were not relevant to the final figure. **d** Molecular characterisation of *Lr34* gene sequences amplified from genomic DNA of Bansi *Lr34*-13-1. Coding sequences of the *Lr34* gene were amplified as five separate fragments (blue lines) using the primer sequences indicated at the end of each amplification product. Each amplification product was sequenced using primers depicted as black arrows with sequence direction shown as hatched black lines. All exons were sequenced in their entirety. Primer sequences and their full names are shown in Table S1, while abbreviated names for primers are used in this figure (colour figure online)

FISH and GISH analyses were then undertaken on BC_1_F_3_ plants 97-14-3 and 97-33-1 which are homozygous for 7DS introgressions derived from plants 97-14 and 97-33, respectively. Upon hybridisation with *Ae. tauschii* (DD) genomic DNA as a probe, small chromosome 7D introgressions were clearly identified (Fig. 5a). Subsequent FISH analysis demonstrated that these 7D introgressions were in chromosome 7A in each case. Therefore, plants 97-14-3 and 97-33-1 were each homozygous for a single distal 7DS introgression in chromosome 7A, consistent with molecular marker analysis undertaken on sibs of these plants described above.

**Fig. 6:**
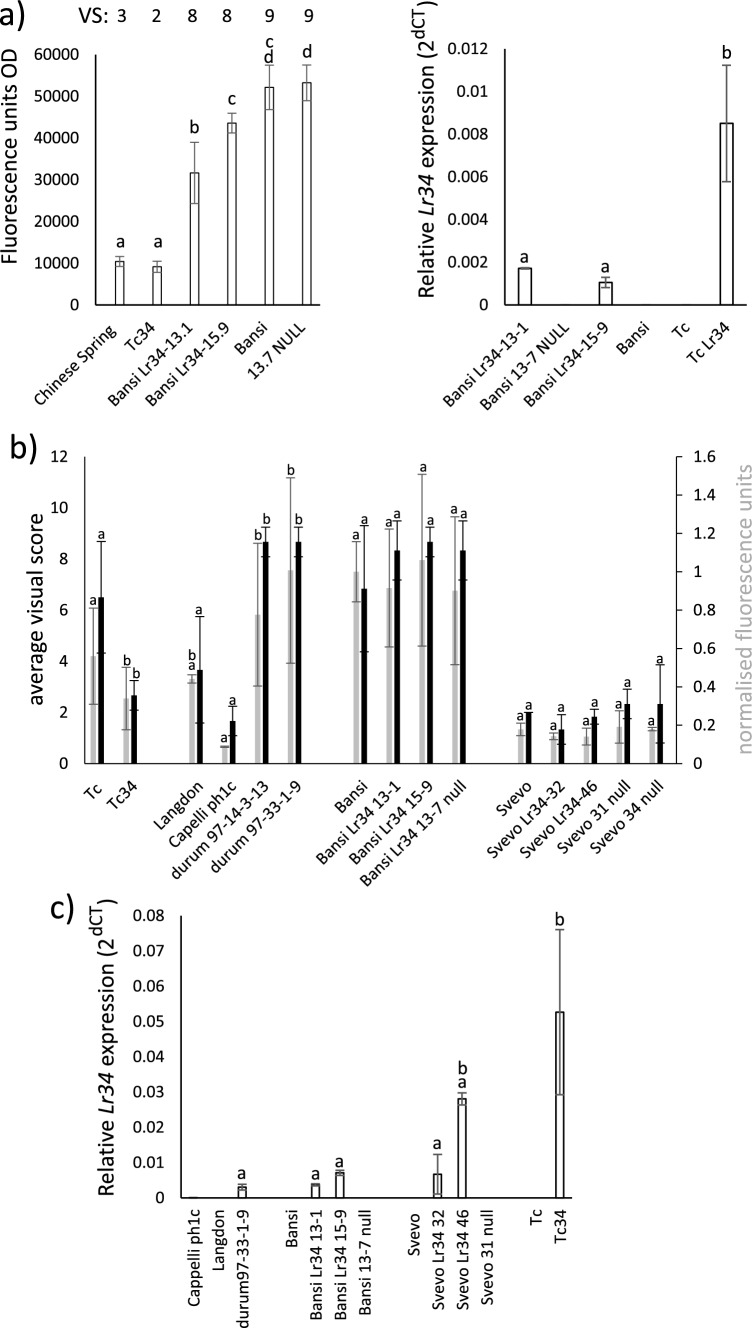
Field trial and expression analysis of *Lr34* durum plants. **a** The graph at left shows fungal biomass quantification of flag leaves from field grown (2021) durum and bread wheat plants. The Y-axis shows fluorescence units with increasing fluorescence equal to increasing fungal biomass. The numbers at the top of the graph indicate visual scores (VS) assigned to each genotype for stripe rust response, on a 0 (immune) to 9 (fully susceptible) scale. The same tissue was used for both biomass quantification and stripe rust response assessment. RNA was also extracted from flag leaves of the same material and *Lr34* expression determined by qRT-PCR (graph on the right). Common letters above columns indicate no significant difference between values (ANOVA with posthoc Tukey). **b** Stripe rust response scores (left hand axis, black columns) and fungal biomass quantification (right hand axis, grey columns) of wheat genotypes grown in 2022. The same field grown material was used for both tests. Common letters above columns indicate no significant difference between values (ANOVA with posthoc Tukey test, *p* < 0.1). Note that statistical comparisons were made only between plants within each of the following groups: [Tc, Tc34], [LDN, Cappelli *ph1c*, durum 97 plants] and [Svevo, Svevo BC plants]. These three groups were not intercompared given their different genetic backgrounds. Biomass data and visual pathology data were compared separately. **c** Quantitative RT-PCR analysis of *Lr34* expression in wheat plants grown in the 2022 field season. Expression levels are relative to the wheat *GAPDH* gene. Expression analysis was undertaken on tissue isolated from the same plants used in (B) above. Samples with common letters above each column are not significantly different (ANOVA, post-hoc Tukey tests)

Plant 97-14-3 was crossed using Bansi as a recurrent parent for 3 generations and segregation of the 7D/7A *Lr34* gene was apparent in progeny derived from selfed BC_2_ Bansi plants, Bansi *Lr34*-13 and Bansi *Lr34*-15, by DNA blot (Fig. 5b, c). Two plants homozygous for the *Lr34* D chromosome introgression in this generation, Bansi *Lr34*-13-1 and Bansi *Lr34*-15-9, and a null sib (Bansi *Lr34*-13-7 null) were selected for progeny field testing in 2021. Visual scoring showed that the progeny of Bansi *Lr34*-13-1 and Bansi *Lr34*-15-9 were susceptible as was the Bansi parent (Fig. 6a). These visual scores were confirmed on the same field material using a chitin-based fungal biomass assay (Fig. 6a). The *Lr34* genotypes of this material was also reconfirmed by using *Lr34* specific markers on tissue taken from the field plots (Fig. S2).

The *Lr34* gene carried by LDN 7D(7A) was originally derived from bread wheat cultivar Chinese Spring (Joppa and Williams 1988). To ensure an equivalent, functional version of the gene was present in the Bansi durum material the *Lr34 ORF* was PCR amplified and sequenced from genomic DNA of line Bansi *Lr34*-13-1. The coding regions of the gene were amplified from this durum line as five discrete fragments and sequenced (Fig. 5d). All exon sequences were identical to the published Chinese Spring sequence (GenBank *FJ436983.1*) as were the intervening intron sequences present in these fragments.

These Bansi families were retested in 2022 along with homozygous families carrying introgressions derived from two Cappelli/LDN 7D(7A) plants, 97-14 and 97-33, and two Svevo *Lr34* lines, Svevo *Lr34*-32 and Svevo *Lr34*-46. Svevo *Lr34* lines were produced by crossing plant 97-33-1 to Svevo and backcrossing for 6 generations, followed by the selection of homozygous plants from a selfed family of a BC6 plant. These Svevo *Lr34* lines contain a smaller 7D introgression derived from plant 97-33, compared with Bansi *Lr34* lines and other 97-14 derived material (Fig. 4). Visual scoring and chitin biomass assays again showed no evidence of resistance in Bansi *Lr34* material nor was resistance apparent in progeny of *Lr34* carrying Cappelli/LDN 7D(7A) plants 97-14-3-13 and 97-33-1-9 compared with the parental controls (Fig. 6b). In contrast, Svevo *Lr34* and control Svevo lines showed high levels of resistance (Fig. 6b) indicating that Svevo contains pre-existing resistance to these *Pst* isolates that masked any potential resistance conferred by the 7DS segment. Accelerated leaf tip necrosis was not observed in *Lr34* durum lines compared with controls.

The absence of *Lr34* resistance in these durum materials was further investigated by expression analysis. RNA was extracted from flag leaves of the infected field materials grown in 2021 and quantitative RT-PCR undertaken using primers specific for the *Lr34* resistance allele. An obvious, four–fivefold reduction in *Lr34* expression was observed in Bansi *Lr34* durum plants when compared with hexaploid bread wheat Thatcher *Lr34* plants, grown under the same field conditions (Fig. 6a). An RNA expression analysis was also undertaken using leaf material from the 2022 field trial and again Thatcher *Lr34* lines showed significantly higher expression of *Lr34* compared with Bansi or Cappelli/LDN 7D(7A) derivatives containing this APR gene (Fig. 6c). These data demonstrate a significantly lower level of *Lr34* expression in these durum plants in the field when compared with the endogenous gene in bread wheat.

## Discussion

There are limited examples of transfer of genes from the D genome of *T. aestivum* to *T. turgidum*. These examples include the introduction of seed storage protein genes for improved dough quality (Lukaszewski and Curtis 1994; Joppa et al. 1998; Ceoloni et al. 1996; Liu et al. 1996; Lukaszewski 2003), the *KNa1* gene for improved Na^+^ tolerance (Dvorak and Gorham 1992; Luo et al. 1996), the *TaAlmt1* gene for improved Al^3+^ tolerance (Han et al. 2014, 2016) and random D-genome fragments (Eberhard et al. 2010; Othmeni et al. 2019). These studies used a mixture of cytogenetics, protein markers, RFLP markers, SNP markers, PCR markers, DArT markers or *Dgas44* qPCR, for identifying and defining introgressions.

Here we used three approaches to identify and define chromosome 7D introgressions into chromosome 7A. Each approach had specific limitations in that KASP and PCR marker analysis could not identify all 7D introgressions due to the limited marker coverage available at the time, 90 K SNP analysis required extensive bioinformatic analysis and cytogenetic analysis resulted in coarse breakpoint refinement. None the less, a combination of these approaches unambiguously identified small, single 7D introgressions in chromosome 7A that encode *Lr34,* with the introgression present in plant 97-33-1 estimated to account for less than 8% of chromosome 7D.

Field trials showed no enhanced adult plant resistance to *Pst* in Cappelli/LDN 7D(7A) or Bansi durum plants carrying *Lr34* on the introgressed 7D/7A segment. *Lr34* expression in flag leaves of field grown Cappelli/LDN 7D(7A) and Bansi durum plants was four–fivefold lower than that observed in hexaploid Thatcher-*Lr34* plants grown in the same field plots. Presumably this low level of expression accounted for the inability of *Lr34* to function in these durum plants and to provide stripe rust resistance. These *Lr34* carrying durum lines have not been challenged with either leaf rust, stem rust, or powdery mildew. Therefore, their potential resistance to these other diseases remains unknown.

There is an established correlation between *Lr34* expression levels and resistance. For example, increased *Lr34* expression occurs in adult plant tissues of bread wheat when compared with seedlings, which presumably accounts for the resistance being observed in mature tissues, only (Krattinger et al. 2009). In addition, bread wheat flag leaf tips show higher expression than do flag leaf bases which also corelates with increased disease susceptibility of the latter tissue (Krattinger et al. 2009). Similarly, *Lr34* resistance to *P. triticina* can be observed in bread wheat seedlings when grown at low temperature (10 °C), which also correlates with higher levels of *Lr34* expression (Rinaldo et al. 2017). As already noted, *Lr34* mediated resistance in transgenic durum wheat was also strongly correlated with transgene expression levels (Rinaldo et al. 2017) as were *Lr34* pleotropic phenotypes in heterologous host species (Risk et al. 2013; Krattinger et al. 2016; Schnippenkoetter et al. 2017; Sucher et al. 2017).

Suppression of wheat disease resistance genes in new genetic backgrounds is not uncommon, particularly when resistance genes are transferred from diploid and tetraploid species to bread wheat (Hiebert et al. 2020 and references therein). Recently, one example was shown to be due to a D genome gene, *Med15*, which encodes a subunit of the transcriptional coactivation mediator complex, that altered gene transcription and suppressed plant immunity (Hiebert et al. 2020). Genetic background was also shown to affect the efficacy of the *Fhb1* Fusarium head blight resistance gene when introgressed into durum wheat from bread wheat (Kirana et al. 2023), while the rye *Pm8* powdery mildew resistance gene is suppressed by the orthologous wheat *Pm3* gene via a post-translational mechanism (McIntosh et al. 2011; Hurni et al. 2014). Recent genomic and transcriptomic analyses have also shown that genes present on introgressed segments in bread wheat can have highly variable expression patterns. In bread wheat plants containing chromosome segments from *Ambylopyrum muticum,* less than half the introgressed genes were expressed (Coombes et al. 2022). Introgressions from *Ae. longissima* were shown to change expression of approximately 4% of endogenous wheat genes located elsewhere in the genome (Dong et al. 2020). Similarly in rice, changes in DNA methylation and gene expression were associated with homoeolog exchange in allotetraploid plants (Li et al. 2019). The interplay between endogenous and homoeologous plant chromatin segments is therefore complex.

The basis for the suppressed *Lr34* expression observed here for two independent 7D/7AS introgressions in durum wheat is unknown. Clearly useful genes present in alien introgressions do not always show suppressed expression. Since E.R. Sears (1956), pioneered interspecies introgressions into wheat there have been numerous introgressions into durum (see above) and bread wheat that provide useful agronomic traits, particularly for disease resistance (Rather et al. 2017; Baranwal 2022). In fact, “linkage drag” caused by expression of additional unwanted genes present on introgressed segments can often be problematic. However, in this study, the *Lr34* APR trait of interest is not manifest in the durum wheat backgrounds examined due to suppressed gene expression.

### Supplementary Information

Below is the link to the electronic supplementary material.

Supplementary file 1 (PPTX 1436 kb)

Supplementary file 2 (XLSX 13 kb)

## Data Availability

All data and materials are available upon request.
