# Supplementary material for: Introgression of the bread wheat D genome encoded Lr34/Yr18/Sr57/Pm38/Ltn1 adult plant resistance gene into Triticum turgidum (durum wheat)
Source: Theor Appl Genet. 2023 Oct 17;136(11):226. doi: 10.1007/s00122-023-04466-z (PMC10581953; doi:10.1007/s00122-023-04466-z)
Supplement: Supplementary file 1 — (PPTX 1436 kb) [file 122_2023_4466_MOESM1_ESM.pptx]

## Slide 1
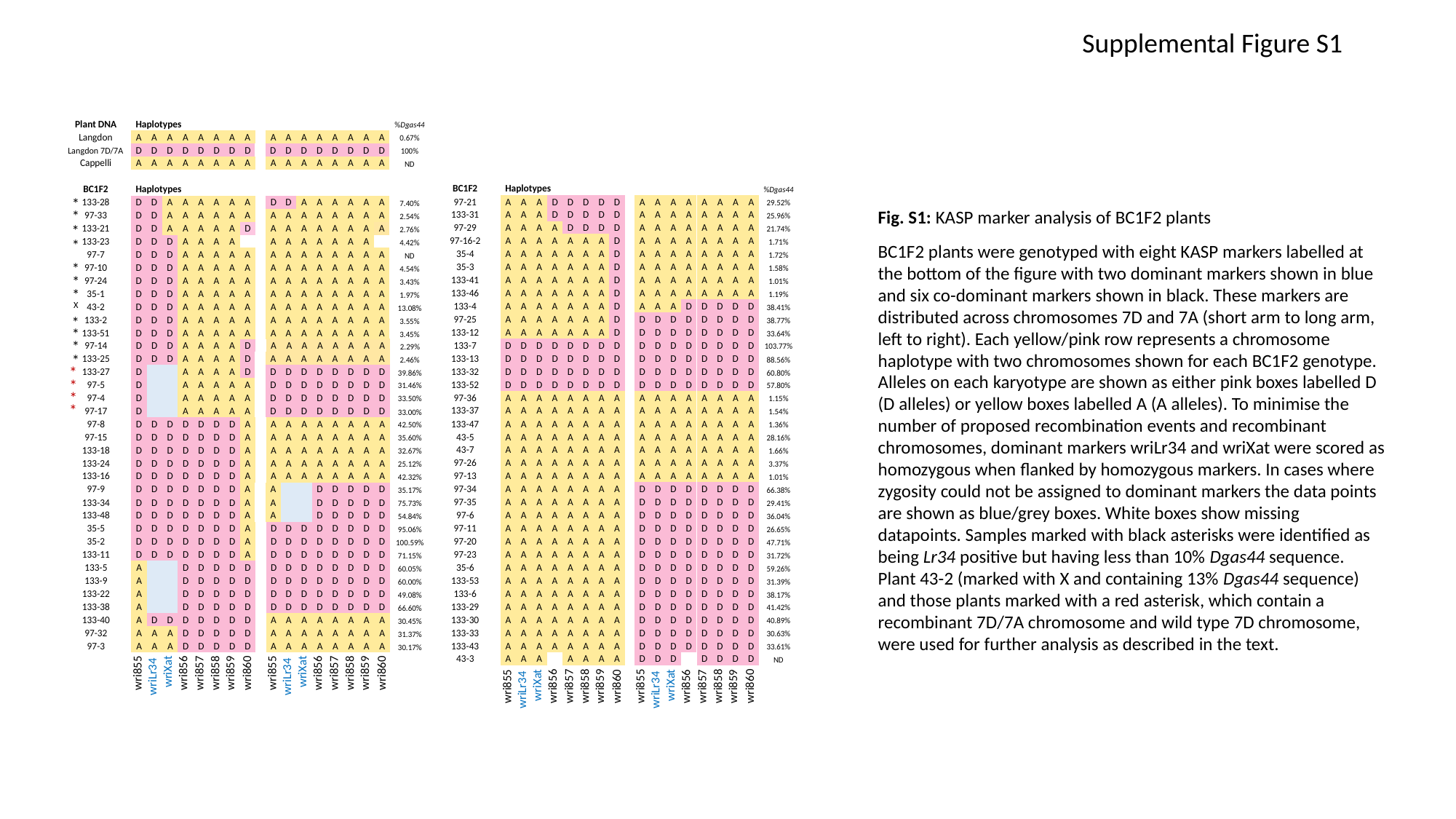

Supplemental Figure S1
wri855
wriLr34
wriXat
wri856
wri857
wri858
wri859
wri860
wri855
wriLr34
wriXat
wri856
wri857
wri858
wri859
wri860
*
*
*
*
*
*
*
X
*
*
*
*
*
*
*
*
wri855
wriLr34
wriXat
wri856
wri857
wri858
wri859
wri860
wri855
wriLr34
wriXat
wri856
wri857
wri858
wri859
wri860
Fig. S1: KASP marker analysis of BC1F2 plants
BC1F2 plants were genotyped with eight KASP markers labelled at the bottom of the figure with two dominant markers shown in blue and six co-dominant markers shown in black. These markers are distributed across chromosomes 7D and 7A (short arm to long arm, left to right). Each yellow/pink row represents a chromosome haplotype with two chromosomes shown for each BC1F2 genotype. Alleles on each karyotype are shown as either pink boxes labelled D (D alleles) or yellow boxes labelled A (A alleles). To minimise the number of proposed recombination events and recombinant chromosomes, dominant markers wriLr34 and wriXat were scored as homozygous when flanked by homozygous markers. In cases where zygosity could not be assigned to dominant markers the data points are shown as blue/grey boxes. White boxes show missing datapoints. Samples marked with black asterisks were identified as being Lr34 positive but having less than 10% Dgas44 sequence. Plant 43-2 (marked with X and containing 13% Dgas44 sequence) and those plants marked with a red asterisk, which contain a recombinant 7D/7A chromosome and wild type 7D chromosome, were used for further analysis as described in the text.

## Slide 2
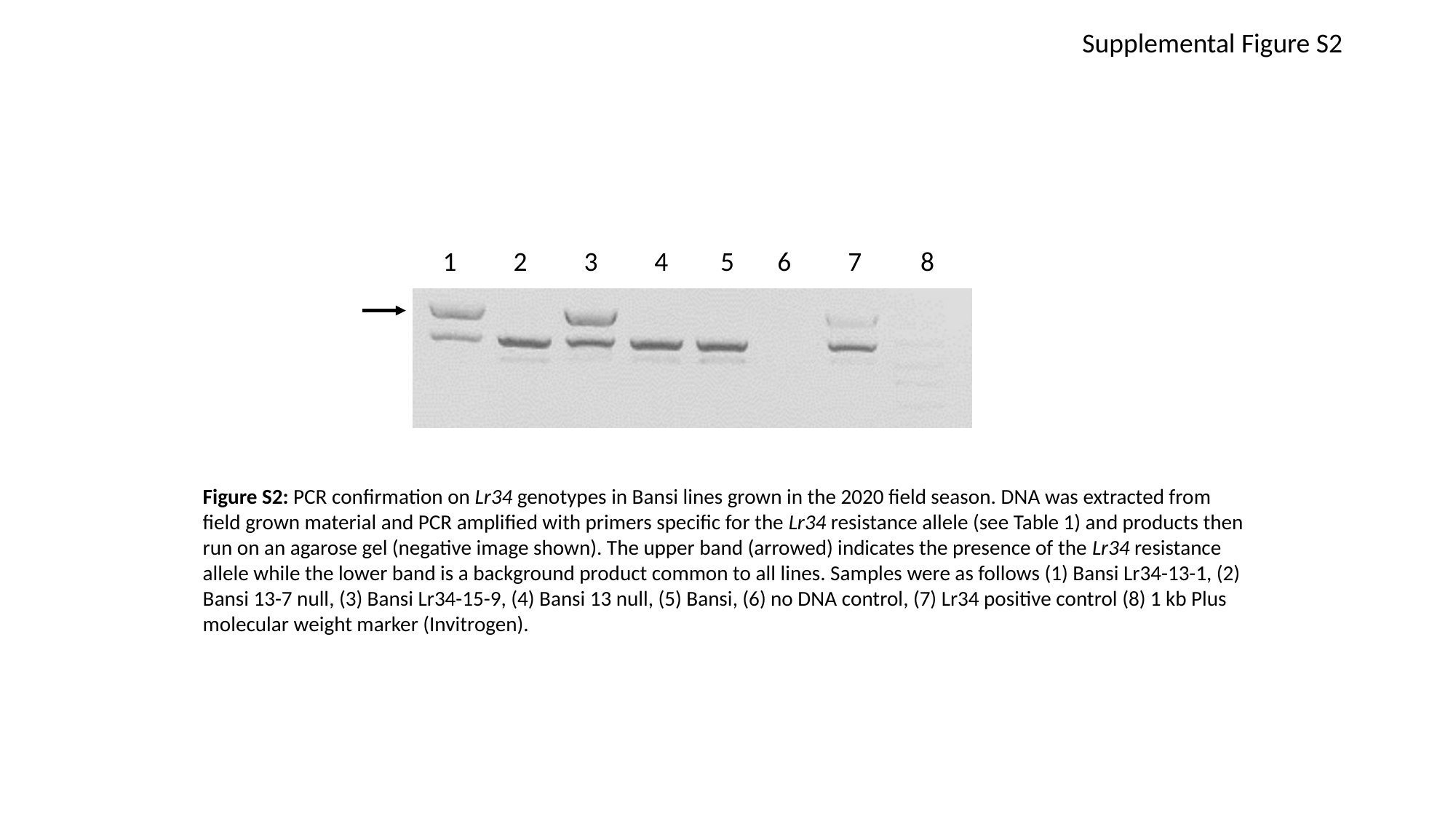

Supplemental Figure S2
4
5
6
7
8
1
2
3
Figure S2: PCR confirmation on Lr34 genotypes in Bansi lines grown in the 2020 field season. DNA was extracted from field grown material and PCR amplified with primers specific for the Lr34 resistance allele (see Table 1) and products then run on an agarose gel (negative image shown). The upper band (arrowed) indicates the presence of the Lr34 resistance allele while the lower band is a background product common to all lines. Samples were as follows (1) Bansi Lr34-13-1, (2) Bansi 13-7 null, (3) Bansi Lr34-15-9, (4) Bansi 13 null, (5) Bansi, (6) no DNA control, (7) Lr34 positive control (8) 1 kb Plus molecular weight marker (Invitrogen).

## Slide 3
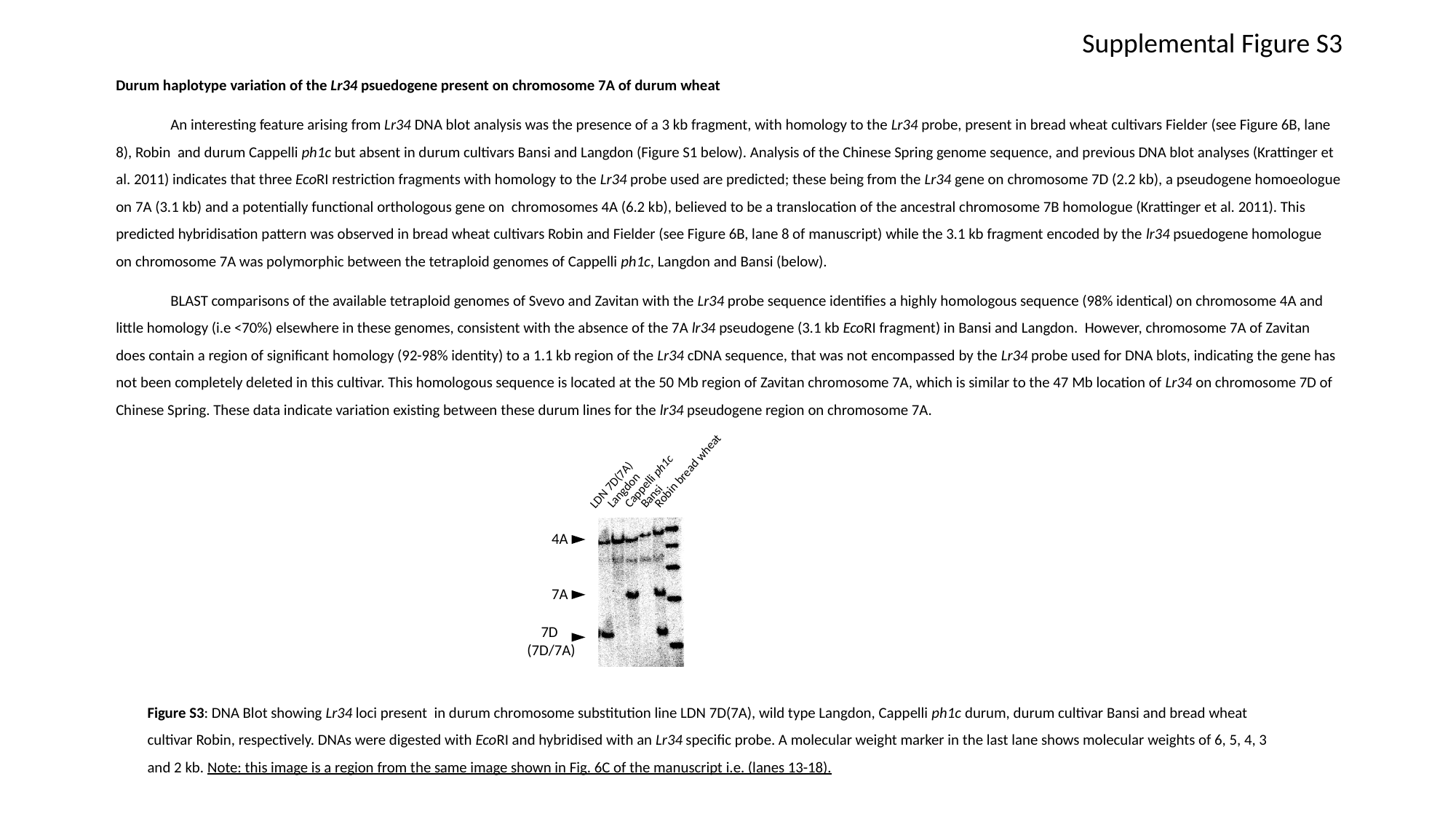

Supplemental Figure S3
Durum haplotype variation of the Lr34 psuedogene present on chromosome 7A of durum wheat
An interesting feature arising from Lr34 DNA blot analysis was the presence of a 3 kb fragment, with homology to the Lr34 probe, present in bread wheat cultivars Fielder (see Figure 6B, lane 8), Robin and durum Cappelli ph1c but absent in durum cultivars Bansi and Langdon (Figure S1 below). Analysis of the Chinese Spring genome sequence, and previous DNA blot analyses (Krattinger et al. 2011) indicates that three EcoRI restriction fragments with homology to the Lr34 probe used are predicted; these being from the Lr34 gene on chromosome 7D (2.2 kb), a pseudogene homoeologue on 7A (3.1 kb) and a potentially functional orthologous gene on chromosomes 4A (6.2 kb), believed to be a translocation of the ancestral chromosome 7B homologue (Krattinger et al. 2011). This predicted hybridisation pattern was observed in bread wheat cultivars Robin and Fielder (see Figure 6B, lane 8 of manuscript) while the 3.1 kb fragment encoded by the lr34 psuedogene homologue on chromosome 7A was polymorphic between the tetraploid genomes of Cappelli ph1c, Langdon and Bansi (below).
BLAST comparisons of the available tetraploid genomes of Svevo and Zavitan with the Lr34 probe sequence identifies a highly homologous sequence (98% identical) on chromosome 4A and little homology (i.e <70%) elsewhere in these genomes, consistent with the absence of the 7A lr34 pseudogene (3.1 kb EcoRI fragment) in Bansi and Langdon. However, chromosome 7A of Zavitan does contain a region of significant homology (92-98% identity) to a 1.1 kb region of the Lr34 cDNA sequence, that was not encompassed by the Lr34 probe used for DNA blots, indicating the gene has not been completely deleted in this cultivar. This homologous sequence is located at the 50 Mb region of Zavitan chromosome 7A, which is similar to the 47 Mb location of Lr34 on chromosome 7D of Chinese Spring. These data indicate variation existing between these durum lines for the lr34 pseudogene region on chromosome 7A.
Robin bread wheat
Cappelli ph1c
LDN 7D(7A)
Langdon
Bansi
4A
7A
 7D
(7D/7A)
Figure S3: DNA Blot showing Lr34 loci present in durum chromosome substitution line LDN 7D(7A), wild type Langdon, Cappelli ph1c durum, durum cultivar Bansi and bread wheat cultivar Robin, respectively. DNAs were digested with EcoRI and hybridised with an Lr34 specific probe. A molecular weight marker in the last lane shows molecular weights of 6, 5, 4, 3 and 2 kb. Note: this image is a region from the same image shown in Fig. 6C of the manuscript i.e. (lanes 13-18).
